# Chronic Lymphocytic Inflammation with Pontine Perivascular Enhancement Responsive to Steroids May Extend above and below Pons and Is Associated with Other Autoimmune Diseases

**DOI:** 10.3390/life11111120

**Published:** 2021-10-21

**Authors:** Brent Berry, Stephanie Joppa, Edward Labin, Vikram Puram, Kaci McCleary, H. Brent Clark, Flavia Nelson

**Affiliations:** 1 Mayo Clinic, Department of Neurology, Rochester, MN 55455, USA; brentb@umn.edu; 2 Department of Electronics, Telecommunications and Informatics, Gdańsk Tech, 80-233 Gdansk, Poland; 3 Department of Internal Medicine, University of Minnesota, Minneapolis, MN 55455, USA; joppa@umn.edu; 4 Department of Neurology, University of Minnesota, Minneapolis, MN 55455, USA; labin017@umn.edu (E.L.); mccl0374@umn.edu (K.M.); fnelson@umn.edu (F.N.); 5 Medical School, University of Minnesota, Minneapolis, MN 55455, USA; 6 Department of Laboratory Medicine and Pathology, University of Minnesota, Minneapolis, MN 55455, USA; clark002@umn.edu

**Keywords:** CLIPPERS, brainstem, perivascular infiltration, neuroinflammation, autoimmune disease

## Abstract

Many autoimmune diseases can affect the central nervous system, and their varying clinical presentations often confound a straightforward diagnosis. In this report, we describe a unique presentation of CLIPPERS syndrome. To our knowledge, this is the first case to demonstrate significant supratentorial involvement with symmetric and non-confluent lesions in the medial orbitofrontal cortex; additionally, this is the second case to describe an association between diagnoses of hypothyroidism and CLIPPERS.

## 1. Introduction

CLIPPERS is a central nervous system (CNS) inflammatory disease affecting the brainstem, cerebellum, and spinal cord, which was defined in 2010 by Pittock et al. [1]. Clinical diagnostic criteria [2] are as follows:

(a) subacute pontocerebellar dysfunction,
(b) CNS symptoms responsive to corticosteroid therapy,
(c) absence of peripheral nervous system disease, and
(d) lack of an alternative explanation.

Imaging criteria include the following:

(a) homogeneous, gadolinium-enhancing nodules without ring enhancement or mass effect predominating in the pons and cerebellum, measuring <3 mm in diameter;
(b) marked improvement with corticosteroid treatment;
(c) homogeneous T2 signal abnormality; and
(d) spinal cord lesions with similar T2- and gadolinium-enhancing lesions.

Neuro-pathological criteria include the following:

(a) dense lymphocytic inflammation with perivascular predominance and parenchymal diffuse infiltration,
(b) T-cells predominating infiltration (CD4 > CD8) with variable macrophage components,
(c) absence of myelin loss or focal secondary myelin loss, and
(d) lack of alternative better explanation. Cerebrospinal fluid is usually noncontributory.

The presence of perivascular and parenchymal inflammatory cell infiltrates and a clinical response to immunosuppression implies an autoimmune or other inflammatory-mediated pathogenesis. The location of the inflammatory infiltrates suggests that the target autoantigen is likely to be located in perivascular regions. Pittock et al. suggested that a diagnosis of CLIPPERS can be made without a brain biopsy if clinical and MRI features of the disease are present and if alternative diagnoses are excluded. Corticosteroid responsiveness is generally prompt with a significant clinical and radiographic response. Relapses typically occur when steroids are withdrawn or tapered below 20 mg PO daily. Patients usually require long-term maintenance immunosuppressive agents. Since the initial report in 2010 by Pittock et al., tens of new cases have been published [1,2]. We report a further patient who adds to those few published cases with supratentorial involvement (contrast enhancing and with predominance in the medial orbitofrontal areas), and an association with hypothyroidism (which would represent the second such association in the literature).

There has been one case of CLIPPERS reported with near-simultaneous diagnosis of Hashimoto’s thyroiditis. It is not clear to us that CLIPPERS has been associated with any other autoimmune conditions.

## 2. Case Presentation

A 62-year-old male with a history of newly diagnosed hypothyroidism presented with progressive neurologic symptoms over 3–5-months, characterized by cranial nerve and cerebellar deficits. Symptoms began with nausea without vomiting, poor appetite from food smelling foul to him, and anorexia, without significant weight loss initially. His symptoms progressed over the following month, and he also developed disequilibrium, constipation, and increased urination, prompting him to be seen by his primary physician who diagnosed hypothyroidism and initiated supplementation with levothyroxine. His symptoms worsened after starting levothyroxine and he thought there was a causal relationship resulting in him discontinuing the medication. His symptoms continued to progress with worsening disequilibrium, gait instability, and paresthesias (top of head, left side of face, thumb and index of left hand, over distal sacrum). One month prior to presentation, he had ongoing sensory changes, diplopia, insomnia, hiccups, a “motorboat-like” tinnitus in the right ear, intermittent blurred vision, and worsening gait resulting in two falls. In the days leading up to presentation, the patient’s voice became hoarse, but he had no issues with dysphagia or swallowing. He described his gait as “my legs do not go the way I want them to” and reported no generalized limb weakness or limb sensory changes except for his left hand. He denied fevers/night sweats, headaches, vertigo, respiratory symptoms, diarrhea, or vomiting.

The patient described himself as a healthy individual with few medical conditions and regular annual physician visits. He was a farmer until 2 years prior to presentation, when he transitioned jobs to mowing lawns and more recently to building the operators on electric doors. He denied significant chemical or heavy metal exposure. He did not drink alcohol regularly or use recreational drugs. He was a pheasant-hunting guide for many years and was concerned that he may have Lyme’s disease, which he was tested for and found negative per his report.

Symptoms worsened over the 1–2 weeks prior to original admission, which resulted in evaluation by endocrinology, leading to a non-contrast MRI of the brain, with concerning findings of parenchymal lesions in the posterior fossa as well as other lesions throughout the cerebrum. MRI report described

*“striking areas of pathologic signal which appear related to areas of mild or minimal swelling in the posterior fossa, with smaller numerous lesions in the cerebrum. The above findings are worrisome for rhombencephalitis, which may be seen with listeria, viral infections. Other inflammatory lesions [sic]? such as vasculitis, sarcoid or tuberculosis may be considered. The pattern is also reminiscent of acute disseminated encephalomyelitis. Lymphoma or metastatic disease is considered unlikely, however, contrast may be of value for further evaluation.”*

**Figure 1:**
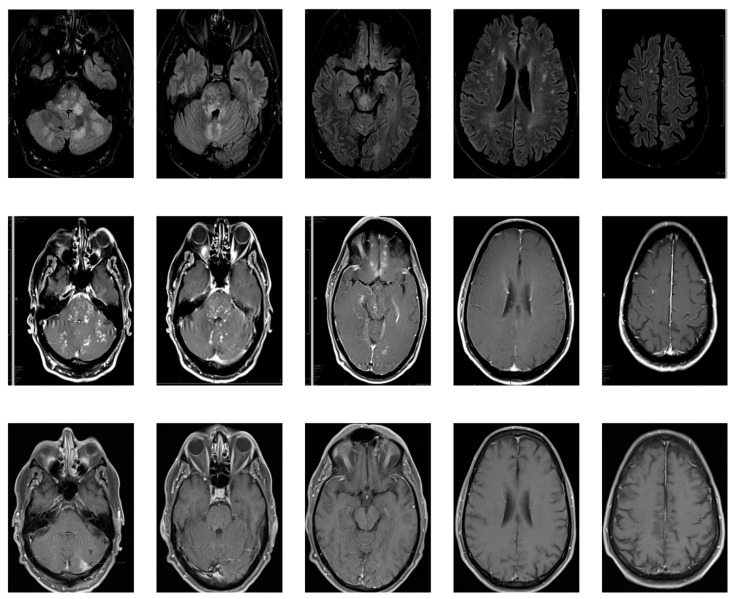
MRI showing Multifocal enhancement of the brain and spinal cord. Striking abnormal patchy and rounded increased T2 signal, predominantly in the brainstem, involving the medulla and the pons, middle, right, and inferior cerebellar peduncles. There is clear involvement of the midbrain and cerebral peduncles. There is involvement of the medial orbitofrontal white matter greater than gray matter and relative sparing of temporal lobes but notably there are supratentorial <3 mm lesions relatively symmetric and some of which contrast-enhance. There is no diffusion restriction. There is minimal mass effect or edema. Row 1: T2 FLAIR; Row 2: T1 post-contrast at presentation; Row 3: T1 post-contrast 20 days after presentation and post-steroids.

**Figure 2:**
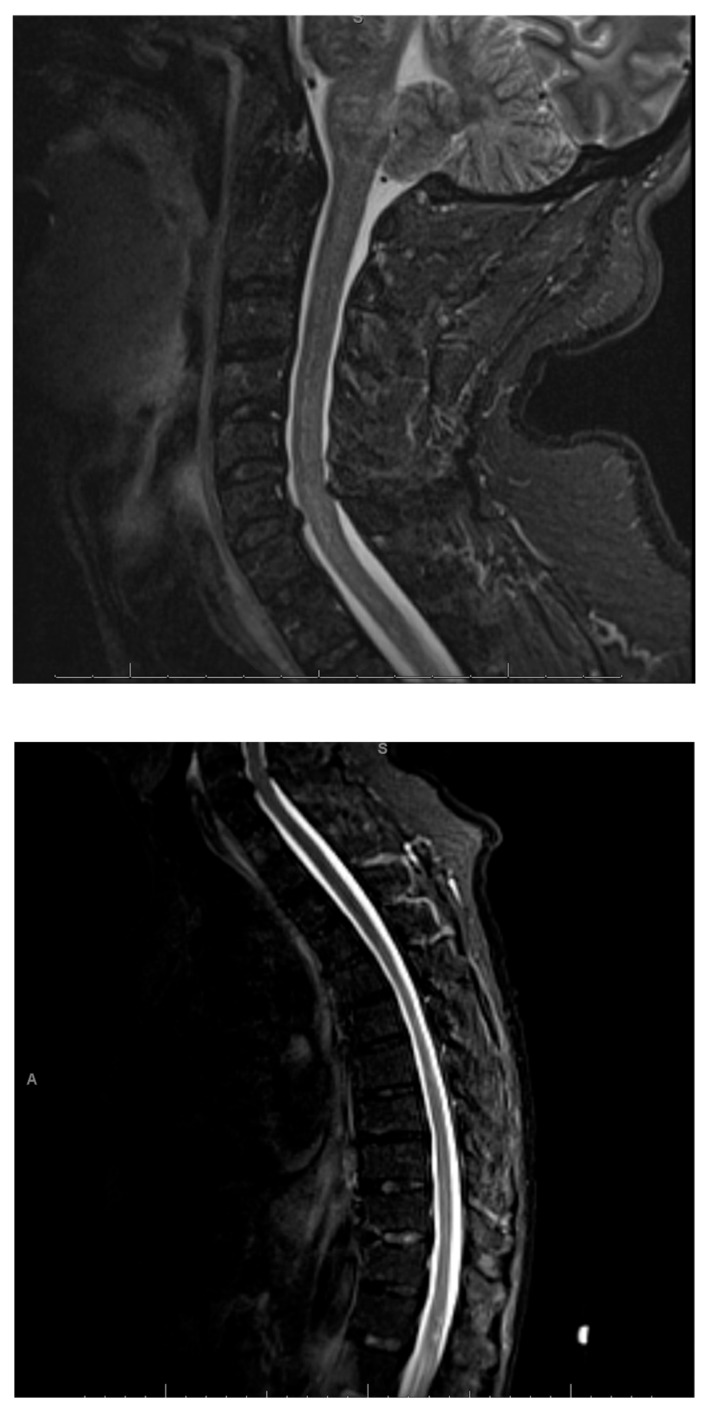
Focal lesions in the distal thoracic spinal cord and portions of the visualized conus medullaris as well as the upper cervical spinal cord.

An MRI with contrast of the entire neuro-axis (Figure 1; Figure 2) was undertaken, which revealed an extensive but patchy increased T2-signal in the cerebellum, brainstem, and forebrain, with mild brainstem swelling. There was patchy enhancement of the hyperintense lesions seen on FLAIR. The cerebral lesions had multifocal areas of enhancement. No overt leptomeningeal enhancement was noted. There was no hydrocephalus, mass effect, or midline shift. In spinal imaging there were patchy foci of T2 hyperintensity and corresponding contrast enhancement in the lower brainstem and cerebellum, extending into the cervico-medullary junction and upper cervical spinal cord.

**Figure 3:**
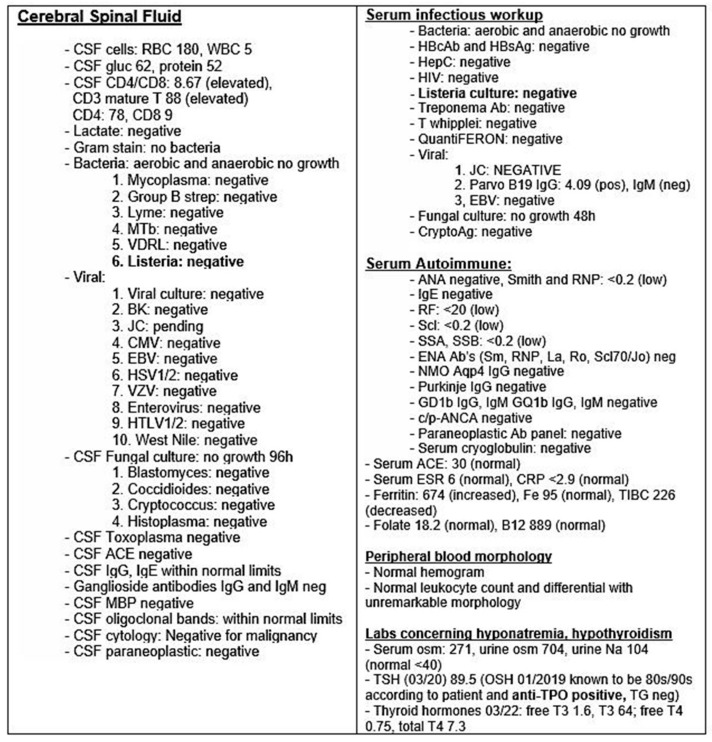
Summary of laboratory findings.

The differential diagnoses considered were multiple sclerosis, neuromyelitis optica spectrum disorder, autoimmune encephalitis, vasculitis, glioma, lymphoma, rhombencephalitis, sarcoidosis, neuro-Behcet’s disease, histiocytosis, and Bickerstaff’s encephalitis. To this end, an extensive laboratory evaluation was undertaken and was found to be unremarkable (Figure 3). Additionally, part of the patient’s workup included a Chest/Abdomen/Pelvis CT scan with contrast that was performed during the patient’s evaluation and revealed only incidental findings of borderline right hilar node and some hypodense liver lesions, which were found on a follow up MRI to represent incidental findings of cysts that were actually decreased in size compared to an outside comparison. Paraneoplastic syndromes were evaluated via Mayo Clinic’s paraneoplastic panel (test code PAC1), which includes AGNA-1, Amphiphysin Ab, ANNA-1, ANNA-2, ANNA-3, CRMP-5-IgG, PCA-1, PCA-2, and PCA-Tr. These were all negative. CSF IgG/Albumin ratio was 0.11 (3.9/36). CSF OCB was 1. Of note, ferritin was elevated, which can indicate nonspecific inflammation and can also be seen in a variety of conditions, including malignancy, metabolic syndrome/diabetes, and infection. For our patient, it was likely multifactorial in the context of inflammatory disorder.

**Figure 4:**
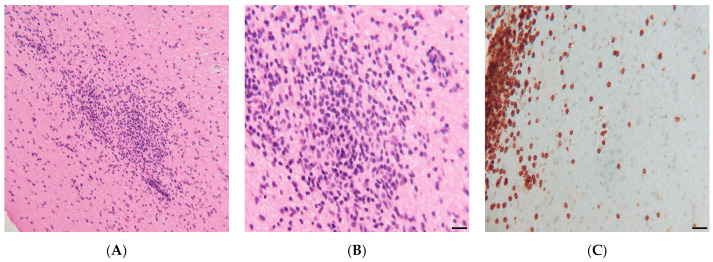
(**A**) Left cerebellar lesion showing inflammatory infiltrates in white matter (hematoxylin and eosin). (**B**) Higher power of lymphocytic infiltrate (hematoxylin and eosin). (**C**) Immunohistochemical stain for CD3 showing T-cell perivascular infiltrate (left margin) and less cellular parenchymal infiltrate in the adjacent white matter.

A stealth-assisted biopsy of the left cerebellar hemisphere was performed. Sections contained several foci of lymphocytic infiltration in the white matter of the cerebellum with sparing of the cortex. Rare histiocytic cells with larger pale nuclei were seen within the dense infiltrates of smaller lymphocytes. There were numerous CD3-positive T-lymphocytes in the perivascular spaces, but also extending with lower density into the surrounding white matter. CD20-staining revealed occasional small B-lymphocytes with no larger CD20-positive cells or atypical cells present. The in situ hybridization for an Epstein–Barr mRNA was negative (Figure 4). Our patient showed some spontaneous improvement; however, following the institution of 60 mg of prednisolone, he responded dramatically and started to walk with improvement of balance, resolution of nystagmus, and significant improvement of coordination. After 2 weeks of prednisolone as an outpatient, he returned to near-normal with some residual disequilibrium. An MRI was repeated at 20 days post initiation of steroids and revealed marked improvement of the lesions in the posterior fossa, spinal cord, and cerebrum, with complete resolution of the lesion in the medial frontal cortex (Figure 1, Row 3). The patient was started on Rituxan maintenance therapy and low dose steroids at 10 mg and remained in remission for at least 20 months at the time of this original report.

## 3. Discussion

Herein, we have described a case of CLIPPERS associated with hypothyroidism and with a novel imaging finding of medial orbitofrontal lobe involvement. This patient’s clinical presentation suggests CLIPPERS, as described by Pittock et al.’s core features [1]. Firstly, the clinical symptoms related directly to brainstem involvement, particularly the subacute gait ataxia (seen in all of Pittock et al.’s patients), and diplopia (seen in one patient). Additionally, the MRI findings were of multiple, small, patchy T2 weighted hyperintensities, with multiple foci of contrast-enhancement, mainly involving the posterior pons and cerebellum as well as the spinal cord. Somewhat uniquely, cerebral features were seen, including in the medial orbitofrontal lobe, an area not previously described in this syndrome. Next, we needed to exclude various other inflammatory, infectious, and paraneoplastic disorders, as well as vasculitis. This work-up was unremarkable except for the already uncovered hypothyroidism with positive TPO Ab. Toxic effect from a number of different sources were considered given the patient’s occupation. However, this was felt less likely over time as the patient did not continue with his previous employment, was in the hospital for some period of time without clear benefit from being away from an offending agent, and notably improved on steroids, which one would not expect except from just a few toxins. Having said that, any of a number of toxins can cause the patient’s presenting symptoms in addition to many others that the patient did not have. Additionally, one would expect most toxins to have effects outside CNS. Metabolic diseases could also be considered and can materialize with the patient’s symptoms, but, again, would not be expected to respond to steroids and, although not impossible, would be less likely without a family history and without presentation earlier in life and the imaging findings would be unusual (usually presenting with confluence of white matter changes in symmetric patterns or there is wide variability depending on the metabolic disease). Ischemic changes may present with a single focal neurologic deficit but not multiple at the same time. Any of a number of autoimmune conditions including multiple sclerosis can present similarly to the patient’s initial report but the imaging findings would be expected to be much different with Dawson finger lesions (radially extending best seen on parasagittal images), juxtacortical and periventricular plaques, which are larger and more discrete than what is seen in CLIPPERS. Paraneoplastic syndromes can present extremely variably and would not necessarily have a characteristic finding. When on the differential, one should evaluate for the primary source and then consider paraneoplastic Ab panel, as was performed in this case. It is actually a mixture of sign/symptoms that can help localize a lesion, but it is taking these with the rest of the data that helps make or break certain diagnoses. The main purpose of a neurologic exam is to localize a lesion; that hypothesis is then tested with modalities such as imaging.

Our patient fulfilled all of these “criteria,” making the diagnosis of CLIPPERS highly probable but we undertook biopsy to exclude, in particular, lymphoma, even though Pittock et al. had treated 50% of their patients without a biopsy.

Our patient had been diagnosed with hypothyroidism and with prior testing was found to be positive for TPO Ab, which is associated with a T-cell-mediated chronic autoimmune thyroid disease. Although the exact mechanism of thyroid tissue destruction is not clear, just as with CLIPPERS, it is considered as a disorder of T-cell-mediated immunity [3]. The coexistence of HT with other non-endocrine autoimmune diseases is well documented. The possibility of Hashimoto’s encephalopathy was considered but felt less likely given focal neurological findings, lack of cognitive disturbance, and lack of seizures, although, admittedly, some of these symptoms are only present in about half of cases [4].

As others have postulated, concurrent detection of two T-cell-mediated autoimmune diseases may not be a happenstance. Moreover, in addition to the highly recommended investigation for underlying lymphomas, as has been suggested [4], in our opinion, patients with CLIPPERS should be also fully interrogated for any other T-cell-mediated immunity issues.

Lesion distribution outside of the pons and adjacent brainstem with extension through the basal ganglia and spinal cord (cervical and thoracic) can be seen [5], and the lesion burden tends to be less the more one moves away from the pons and cerebellum. More widespread areas of involvement have been documented in the literature. Tobin and Pittock describe a series where 61% of patients had lesions outside the cerebellum and pons [6]. Never though has there been description or radiographic illustration of orbital frontal involvement, as in our case [7,8,9,10,11].

Additionally, while many cases have shown documented improvement on non-steroid sparing immune modulating agents such as mycophenolate or azathioprine, our patient remained stable on rituximab for over 20 months until this medication was held to improve the response to COVID-19 vaccination, at which point a severe exacerbation of his syndrome occurred, which, this time, was not responsive to steroids or plasma exchange for several months despite the reintroduction of rituximab. Multiple courses of IV steroids seemed to temporarily help but the stability remained when the prednisone dose was not reduced below 40 mg/day, this was not the case prior to holding rituximab when he seemed stable at the 10 mg/day dose. This experience shows the potential rebound effect that is seen when withholding other highly anti-inflammatory medications for MS such and underscores the importance of high dose steroids to prevent the rebound, which, retrospectively, should have been given.

## 4. Conclusions

CLIPPERS describes a new and often not considered entity affecting the CNS. It is not clearly defined, and remains a debated issue, and as per the British Society for Immunology, whether it represents an independent, actual new disorder or a syndrome that includes etiologically heterogeneous diseases. Clinicians and radiologists should be aware of this condition and its differential diagnoses, given that CLIPPERS constitutes a treatable condition and that patients may benefit from an early introduction of glucocorticosteroid immunosuppression. Our contribution to this work is to further describe the radiologic representation of what is possible with CLIPPERS and also to further knowledge of possible mechanisms by describing an association with hypothyroidism.

## Data Availability

Correspondence with authors can be undertaken regarding the details of this clinical report.
